# Effect of Addition of Tannin Extract from Underutilized Resources on Allergenic Proteins, Color and Textural Properties of Egg White Gels

**DOI:** 10.3390/ijms25074124

**Published:** 2024-04-08

**Authors:** Yoko Tsurunaga, Mika Ishigaki, Tetsuya Takahashi, Shiori Arima, Sae Kumagai, Yoshimasa Tsujii, Shota Koyama

**Affiliations:** 1 Faculty of Human Science, Shimane University, Matsue 690-8504, Shimane, Japan; 2 Institute of Agricultural and Life Sciences, Academic Assembly, Shimane University, Matsue 690-8504, Shimane, Japan; 3 Graduate School of Human and Social Sciences, Shimane University, Matsue 690-8504, Shimane, Japan; 4 Kewpie Research Division for Egg Innovation, Tokyo University of Agriculture, Setagaya City 156-8502, Tokyo, Japan; 5 Faculty of Applied Biosciences, Tokyo University of Agriculture, Setagaya City 156-8502, Tokyo, Japan

**Keywords:** allergenic proteins, color, textural properties, egg white, polyphenol, tannin, chestnut inner skin, young persimmon fruit, bayberry leaf

## Abstract

Tannins, present in numerous plants, exhibit a binding affinity for proteins. In this study, we aimed to exploit this property to reduce the concentration of allergenic egg white proteins. Tannins were extracted, using hot water, from the lyophilized powder of underutilized resources, such as chestnut inner skin (CIS), young persimmon fruit (YPF), and bayberry leaves (BBLs). These extracts were then incorporated into an egg white solution (EWS) to generate an egg white gel (EWG). Allergen reduction efficacy was assessed using electrophoresis and ELISA. Our findings revealed a substantial reduction in allergenic proteins across all EWGs containing a 50% tannin extract. Notably, CIS and BBL exhibited exceptional efficacy in reducing low allergen levels. The addition of tannin extract resulted in an increase in the total polyphenol content of the EWG, with the order of effectiveness being CIS > YPF > BBL. Minimal color alteration was observed in the BBL-infused EWG compared to the other sources. Additionally, the introduction of tannin extract heightened the hardness stress, with BBL demonstrating the most significant effect, followed by CIS and YPF. In conclusion, incorporating tannin extract during EWG preparation was found to decrease the concentration of allergenic proteins while enhancing antioxidant properties and hardness stress, with BBL being particularly effective in preventing color changes in EWG.

## 1. Introduction

Chicken eggs are a prevalent source of food allergies. The “National Survey on Immediate Type Food Allergy” by Japan’s Consumer Affairs Agency in 2011 identified chicken eggs as the leading source, constituting 39.0% of all reported food allergies. Notably, this allergy affects a significant portion of the population, with 57.6% of 0-year-olds and 39.1% of 1-year-olds reporting allergies to chicken eggs [1]. Eggs are widely utilized in cooking due to their nutritional value and versatility. They are commonly used in various food products such as bread, confectionery, processed meat (ham), and seasonings (mayonnaise). The conventional approach to egg allergy management involves eliminating or significantly reducing egg consumption from the diet. However, this restriction poses a substantial challenge to daily life, and the risk of inadvertent exposure remains, potentially leading to severe anaphylactic reactions. Several studies have explored methods to decrease the allergenicity of chicken eggs. The primary allergens in eggs are ovomucoid (OVM) and ovalbumin (OVA), constituting approximately 11% and 54% of the egg white (EW) proteins, respectively [2]. OVA is less allergenic and more degradable when the proteins are denatured by heating [3]. However, OVM is thermostable and does not lose its allergenic activity [4]. While heating can reduce the allergenicity of OVA, OVM remains thermostable and allergenic even after cooking. Research has shown that removing OVM from EW significantly reduces allergenic activity, highlighting its crucial role in egg allergies [5]. Various methods, such as pulsed electrolysis and electrolysis treatment, have been explored to reduce the IgE-binding capacity of egg allergens [6,7]. However, these methods require cost, labor, and effort, and are not systems that can be implemented immediately at processing sites. As described above, egg allergy impacts many people, and hence, reducing the allergenicity of chicken eggs is crucial. Although research is ongoing on this topic, the main method for avoiding the effects of chicken egg allergy is the elimination diet [8]. However, this places a heavy burden on the patient and their family. This study developed a new hypoallergenic method to treat chicken egg allergy. Tsurunaga et al. conducted research on chestnut peels discarded during food processing [9], persimmon fruits discarded during picking and cultivation [10], and bayberry leaves (BBLs) discarded during pruning [11]. The results showed that they contained large amounts of tannins, which are known for having high protein-binding [12] and antioxidant activities, and polyphenols [13]. Tannins can be divided into two fundamentally different chemical structures: hydrolyzable and condensed tannins. Hydrolyzable tannins are polymers of gallic acid and ellagic acid covalently linked to esters. Condensed tannins are ether-covalently bonded polymers of flavan-3-ol. Out of these tannins, condensed tannins bind more strongly to proteins [14], and it has been reported that condensed tannins are abundant in CIS, YPF, and BBLs [15,16,17]. In a previous study, we developed a technique to reduce the allergenicity of processed wheat products by adding tannins to wheat, taking advantage of their protein-binding properties [18].

In this study, we aimed to create a new and simple hypoallergenic method for treating chicken egg allergy and used tannins found in chestnut peels, persimmon fruits, and BBLs (pruned material) due to their known protein-binding and antioxidant properties. This was performed by preparing an EWG by adding tannin extracts to an EWS and evaluating allergen reduction, antioxidant properties, textural properties, and the impact of tannin addition on the inherent coagulability of egg white.

## 2. Results and Discussion

### 2.1. Characteristics of EWS and Tannin Materials Solution

Table 1 presents the total polyphenol content (TPC), soluble tannin content (STC), image, color, pH, and Brix results for raw EWS, CIS, YPF, and BBL solutions. TPC exhibited the order YPF > CIS > BBL > EWS, with EWS surprisingly displaying a high value of 411.5 mg catechin (CTN) eq/100 mL, potentially attributed to the Folin–Ciocalteu reagent reacting with tyrosine, tryptophan, and cysteine residues, resulting in a deep blue color. The STC values were 391.3 ± 13.3, 331.5 ± 15.5, and 322.5 ± 27.9 CTN eq/100 mL for CIS, YPF, and BBL, respectively, with smaller differences between materials than observed for TPC. YPF had a higher L* value, and CIS had higher a* value than the other materials. BBL showcased a higher b* value, indicating a yellowish color, and was more transparent than the CIS and YPF solutions. The pH ranged from 4.0 to 4.8 for CIS, YPF, and BBL, and 7.2 for EWS, indicating neutrality. The Brix values followed the order YPF > CIS > EWS > BBL, with YPF registering as high as 10.1, likely due to its known higher content of carbohydrates, such as fructose, glucose, and sucrose [19].

### 2.2. Evaluation of EW Allergen

#### 2.2.1. Sodium Dodecyl Sulfate-Polyacrylamide Gel Electrophoresis (SDS-PAGE) Analysis

**Figure 1:**
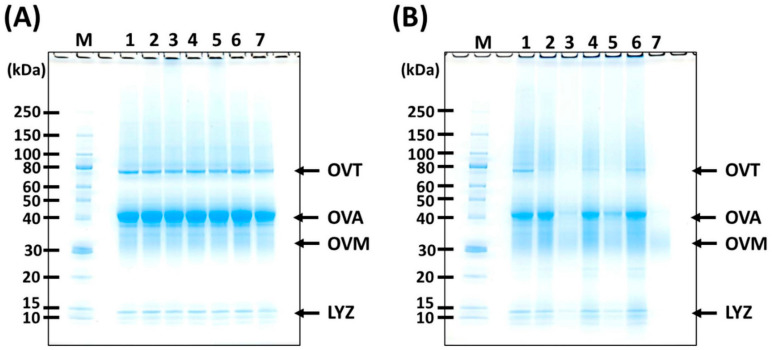
SDS-PAGE patterns of tannin-treated EW proteins. (**A**) EWS; (**B**) EWG. M, molecular mass standards. 1, control; 2, 10% CIS; 3, 50% CIS; 4, 10% YPF; 5, 50% YPF; 6, 10% BBL; 7, 50% BBL. EW, egg white; EWS, egg white solution; EWG, egg white gel; CIS, chestnut inner skin; YPF, young persimmon fruit; BBL, bayberry leaf. OVT, ovotransferrin; OVA, ovalbumin; OVM, ovomucoid; LYZ, lysozyme.

SDS-PAGE was conducted to assess the interactions between EW proteins and tannins, and bands of writing proteins were referenced from previous studies [20]. In the electrophoretic pattern of EWS, distinct bands for ovotransferrin (OVT), OVA, OVM, lysozyme (LYZ), and aggregates within the molecular weight range from 80 to 250 kDa were evident (Figure 1A). No apparent differences were observed in the band patterns of EW proteins with or without the presence of tannin extract. However, a faint band corresponding to aggregates larger than 250 kDa was noticeable in the EWS containing CIS and YPF.

The band patterns of the EWG varied depending on the concentration and type of tannin extract (Figure 1B). Notably, the band intensity of OVT significantly decreased in the EWGs with 10% tannin extracts compared to those without tannin, and no OVT band was observed in the EWG with 50% tannin extracts. Furthermore, the band intensities of OVA, OVM, and LYZ were lower in the EWGs containing 50% CIS and BBL than in those with no tannins or 50% YPF. Specifically, the EWG with 50% BBL displayed no monomeric bands, except for trace amounts of OVM.

Under these experimental conditions, the majority of non-covalent and disulfide bonds were cleaved by the reagents present in the sample buffer. Hence, the observed band attenuation was likely attributed to aggregation via covalent bonds rather than disulfide bonds [20,21]. Some studies have explored the formation of covalent bonds between proteins and tannins [22,23]. Typically, this reaction involves the conversion of the phenolic hydroxyl group of tannins to quinone through alkaline or polyphenol oxidase treatment, followed by addition to the amino acid residues of proteins [22]. According to Pizzi [23], tannins and protein hydrolysates form covalent bonds when heated at 80 °C for 60 min in a neutral solution [23], a condition nearly identical to that in this study. Other potential bonds include lanthionine and lysinoalanine bonds formed between EW proteins during gelation [20]. However, the beta elimination of cysteine, a key reaction for lanthionine or lysinoalanine bonding, was unlikely to be affected by tannins. Thus, during heat-induced gelation, tannins and EW proteins formed aggregates with covalent bonds, indicating a process beyond mere mixing with tannin extracts. Notably, BBL served as a crosslinker that effectively aggregated most EW proteins, including OVA.

#### 2.2.2. Enzyme-Linked Immunosorbent Assay (ELISA)

**Figure 2:**
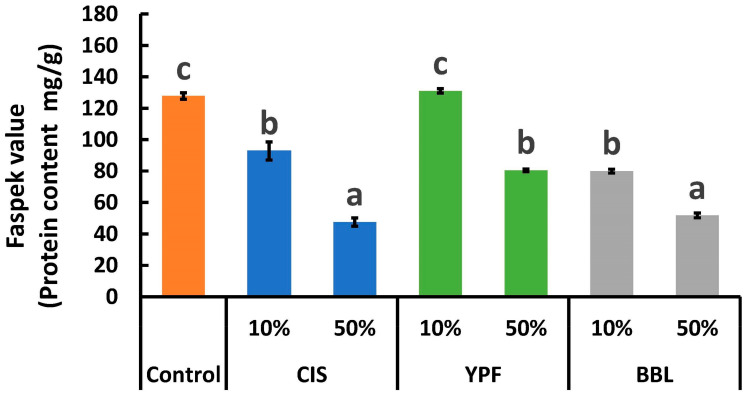
Faspek values of tannin-treated egg white gels. All results were obtained using Tukey’s test for multiple comparisons. Faspek, FASPEK ELISA II^®^ series for egg white albumin. Different letters indicate significant differences at *p* < 0.05. Data are expressed as the mean ± SE (*n* = 4). CIS, chestnut inner skin; YPF, young persimmon fruit; BBL, bayberry leaf.

The protein content of the EWG samples, measured using the FASPEK ELISA II^®^ series for albumin (referred to as Faspek value) (Morinaga Institute of Biological Science, Yokohama, Japan), is illustrated in Figure 2. In comparison to the controls, the Faspek values were significantly reduced in all tannin-supplemented groups (except for the 10% YPF) with more pronounced reductions observed with higher amounts of tannin extract addition (*p* < 0.05). A comparative analysis of the three tannin extracts employed in this study (CIS, YPF, and BBL) revealed that CIS and BBL were more effective than YPF. Hence, the results of the ELISA were found to be consistent with the electrophoresis results. Specifically, the 50% CIS sample demonstrated a value of 47.6 mg/g (62.8% reduction), and the 50% BBL sample exhibited a value of 51.8 mg/g (59.5% reduction), in contrast to the control Faspek value of 127.8 mg/g. Conversely, in the case of the 10% YPF (131.1 mg/g) sample, there was no significant difference from the control value (127.8 mg/g). The 50% YPF (80.5 mg/g), 10% CIS (92.8 mg/g), and 10% BBL (80.0 mg/g) samples displayed comparable Faspek values with no significant differences. OVA constitutes the majority (54% *w*/*w*) of EW [24], and is composed of 385 amino acids with a molecular weight of 45 kDa and a pI of 4.5 [25]. OVA undergoes three-dimensional structural changes through various treatments, including heating [26]. Such changes involve the formation of new disulfide bonds or the addition of side chains, resulting in irreversible modifications, such as aggregation, which are commonly observed during the heat treatment of globular proteins [27]. Conformational changes induced by heat treatment decrease the allergenicity of food proteins by either destroying or shielding specific epitopes or altering protein digestibility [28,29]. For instance, Jiménez-Saiz et al. [30] reported a reduction in the amount of rabbit IgG and human IgE binding to OVA after heating at 90 °C for 15 min. Claude et al. [31] also demonstrated that larger aggregates, when analyzed using sera from egg-allergic patients or sera from OVA-sensitized mice, exhibited lower IgE- and IgG-binding capacities compared to natural OVA. As mentioned, allergy reductions of OVA in EWG through heat treatment have been widely reported. In addition, various other hypoallergenic techniques for eggs have been reported [24]. It has been reported that when egg albumin was treated under pressurized thermal conditions, SDS-PAGE staining spread at 130 °C (0.3 MPa) and no specific bands were observed, and, above 150 °C (0.5 MPa), the staining bands were fuzzy and shifted to the low-molecular-weight side [32], which indicates that heating under normal pressure conditions (100 °C) does not change the OVA content, making hypoallergenization difficult. Another report examined the reduction of egg allergens in cakes containing gamma-irradiated egg whites [33]. It was found that the OVA concentration without gamma radiation was 432.88 mg/g, whereas it was 14.27 and 8.78 mg/g in the 10 and 20 kGy-irradiated samples, respectively, indicating a very high efficacy [33]. However, we believe that its practical application is challenging because special irradiation equipment is required, and the use of radiation for food products may be restricted. Furthermore, a study examined the immunogenic and structural properties of OVA under pulsed-electric-field treatment; when the OVA samples were treated with a high electric-field strength (>25 kV/cm, 180 μs) or for long durations (>60 μs, 35 kV/cm), the results revealed that OVA aggregation significantly reduced the IgG-binding ability, and, in particular, it decreased by approximately 30% after treatment at 35 kV/cm and 180 μs [6]. This technique also requires special equipment. However, we revealed the potential of a new and simple allergen reduction technology for EW by adding tannin extracts. Moreover, this technique facilitates the application of tannin-rich but underutilized resources. Based on these points, the technique of adding tannin extracts is a promising approach; however, further research is required to completely reduce allergens.

### 2.3. Fourier Transform Infrared Spectroscopy (FT-IR)

**Figure 3:**
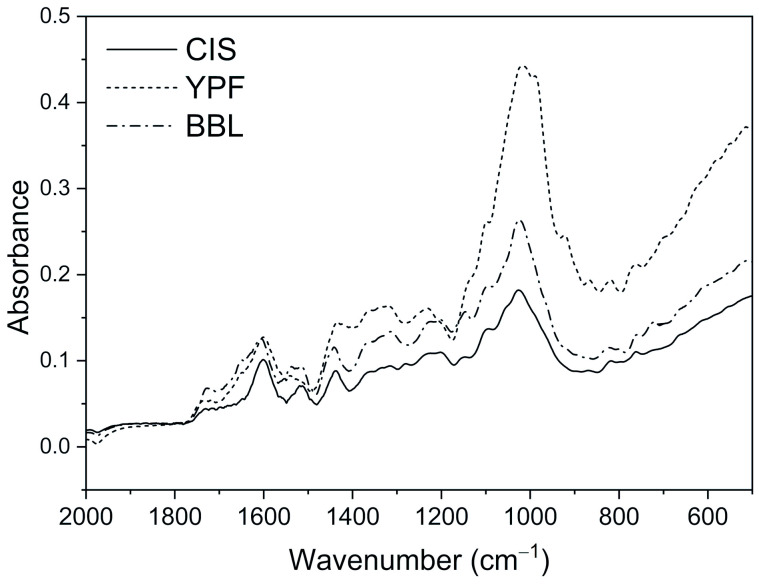
IR absorbance spectra in the 2000–500 cm^−1^ region of three types of tannin. IR, infrared spectroscopy; CIS, chestnut inner skin; YPF, young persimmon fruit; BBL, bayberry leaf.

**Figure 4:**
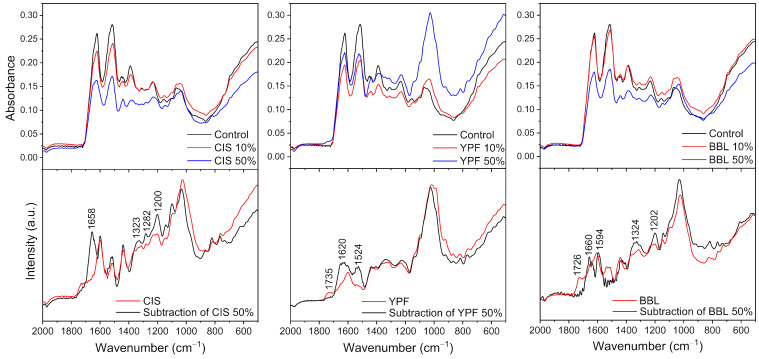
Upper column: comparison of IR absorbance spectra of pure EWG (control) and EWG with CIS, YPF, and BBL at 10 and 50% concentrations. Bottom column: subtraction spectra resulting from the subtraction of the pure EWG spectra from the spectra of the mixed EWG and tannin samples at 50% concentration, and the pure tannins. IR, infrared spectroscopy; EWG, egg white gel; CIS, chestnut inner skin; YPF, young persimmon fruit; BBL, bayberry leaf.

To investigate the structural changes of EWG caused by adding tannins, FT-IR spectroscopic analysis was carried out. Figure 3 depicts the IR absorbance spectra in the 2000–500 cm^−1^ region for three types of tannins. The band assignment for these tannins is summarized in Table 2 [34,35,36]. The spectra show characteristic patterns owing to the molecules with aromatic rings and phenol groups. The spectral variations of the EWG caused by adding three types of tannins are shown in the upper column of Figure 4. The absorbance spectrum of the pure EWG is defined as a control and was compared to the spectra of the mixed samples of tannins at 10% and 50% concentrations. Spectral variations caused by changes in the ratio of the two compositions were clearly observed. To uncover the mechanisms of the intermolecular interactions between the EWG and tannins that induce aggregations, the spectra were analyzed in detail. Namely, we investigated whether the spectral variations could be explained simply by the spectral changes caused by mixing two compositions, or by the further variations caused by the molecular structural changes between the two compositions.

First, the spectrum of the pure EWG was subtracted from that of each mixed sample. The subtracted spectra were expected to show similar patterns to those of the pure tannins added if the molecular structure not changes due to the interaction between the EWG and the tannin molecule. Conversely, if the molecular structure changes due to the interaction between the EWG and the tannin molecule, then the subtracted spectrum is likely to have a different pattern to that of the pure tannin. The bottom column of Figure 4 compares the subtracted spectra for each tannin at 50% concentration with those of each pure tannin. Overall, the subtracted spectra show similar spectral patterns to those of each tannin. However, double bands appeared in the 1600–1500 cm^−1^ region, and the bands at approximately 1320 and 1220 cm^−1^ had remarkably intensified. These variations in the spectra of the mixed samples were highly similar to those observed for the transition of phenol into the quinone–phenolate structure [37]. That is, the results of the spectroscopic analysis were found to be consistent with the electrophoresis results.

### 2.4. Total Polyphenol Content

**Figure 5:**
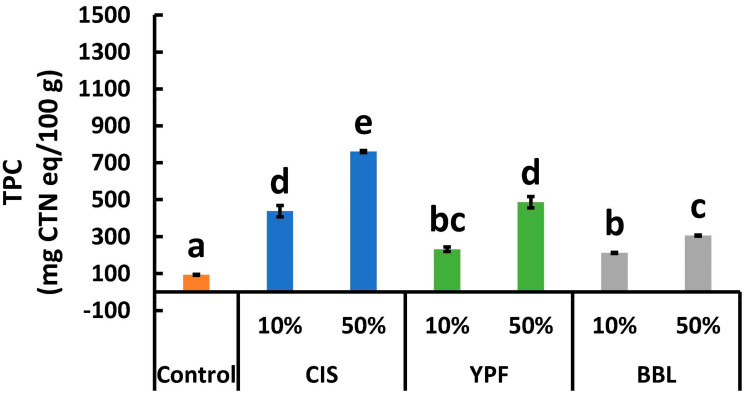
TPC of tannin-treated EWG. All results were obtained using Tukey’s test for multiple comparisons. Different letters indicate significant differences at *p* < 0.05. Data are expressed as the mean ± SE (*n* = 6). TPC; total polyphenol content; CIS, chestnut inner skin; YPF, young persimmon fruit; BBL, bayberry leaf; EWG, egg white gel.

Figure 5 depicts the TPC values of the EWG, which were significantly higher (*p* < 0.05) in all tannin extract-supplemented treatments compared to the control. Specifically, the TPC value of the 10% CIS sample (was 4.7 times (437.3 mg/g) higher than the control (93.1 mg/g), while for the 50% CIS sample, an 8.2 times (760.8 mg/g) increase over the control was recorded. The addition of tannin extract resulted in an EWG with elevated TPC levels (Figure 5) and allergen reduction (Figure 1 and Figure 2). CIS polyphenols are known for their antioxidant and antibacterial effects [9], YPF polyphenols exhibit bile acid-binding activity and hypoglycemic effects in mice [38], and BBL polyphenols have been reported to demonstrate anti-obesity activity in a rat model of high-fat diet-induced obesity [39]. The incorporation of tannin material extract imparts these health functionalities to the EWG, enhancing its health benefits.

### 2.5. Appearance and Color

**Figure 6:**
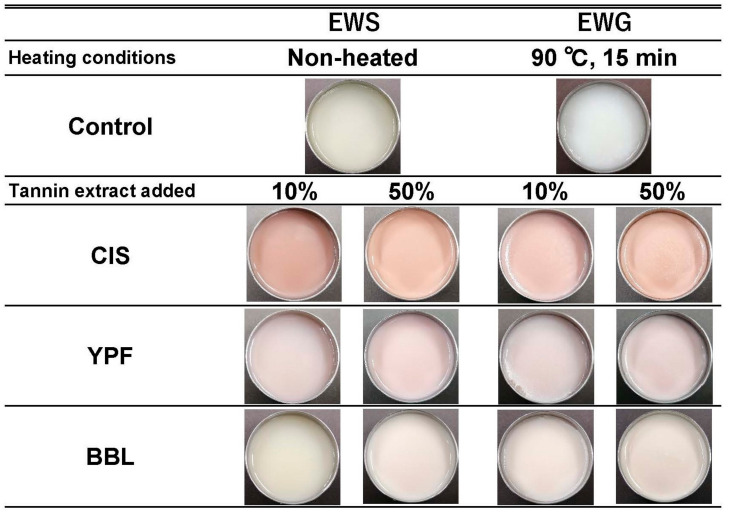
Images of tannin-treated EWS and EWG. EWS, egg white solution; EWG, egg white gel; CIS, chestnut inner skin; YPF, young persimmon fruit; BBL, bayberry leaf.

As depicted in the digital camera image in Figure 6, the addition of YPF and CIS resulted in both the EWS and EWG adopting a darker reddish hue than the control, and this effect became more pronounced with an increased tannin extract addition ratio. This trend was particularly prominent in the case of the CIS sample. In contrast, the BBL sample exhibited minimal change in appearance with the addition of the extract compared to the YPF and CIS samples (Figure 6). The BBL solution, being more transparent than the CIS and YPF solutions (Table 1), likely contributed to the subdued change in the appearance of the EWG. This subtle impact on the EWG’s appearance is a crucial factor to consider when assessing quality.

**Figure 7:**
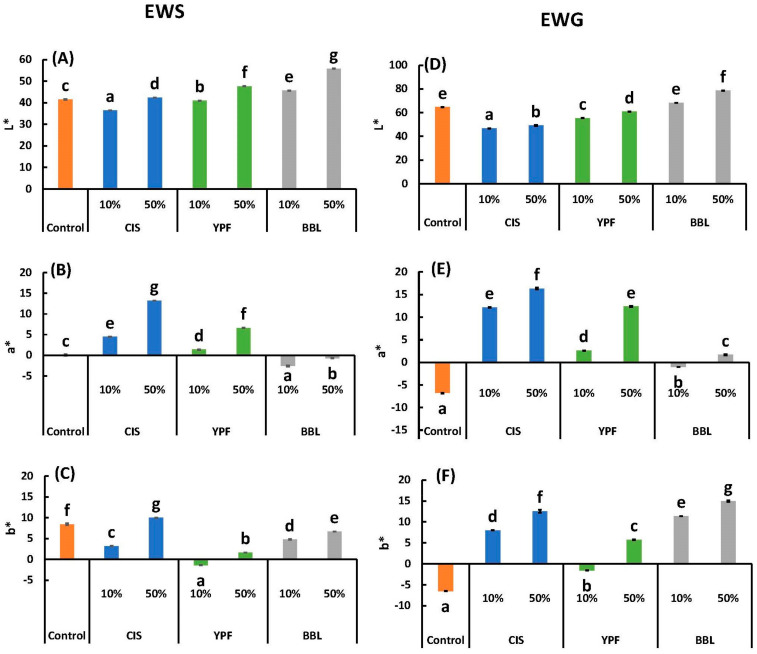
(**A**) L*, (**B**) a*, and (**C**) b* of tannin-treated EWS, and (**D**) L*, (**E**) a*, and (**F**) b* of EWG. Different letters indicate significant differences at *p* < 0.05. Data are expressed as the mean ± SE (*n* = 10). All results were obtained using Tukey’s test for multiple comparisons. EWS, egg white solution; EWG, egg white gel; CIS, chestnut inner skin; YPF, young persimmon fruit; BBL, bayberry leaf.

The L* value signifies lightness, a* indicates the color from green to red, and b* indicates the intensity of the color from blue to yellow. The L*, a*, and b* values for the CIS extract were 39.3 ± 0.0, 14.7 ± 0.0, and 11.6 ± 0.1, respectively; for the YPF extract, they were 49.5 ± 0.1, 12.2 ± 0.1, and 8.9 ± 0.1; and, for the BBL extract, they were 36.6 ± 0.1, 12.9 ± 0.2, and 14.5 ± 0.2 (Table 1). Comparing the effects of adding tannin extract on the L*, a*, and b* values, it was evident that the a* and b* values were conspicuously influenced by the addition of the tannin extract. The broad trends of the L*, a*, and b* values for each treatment were similar for the pre-heated EWS and post-heated EWG (Figure 7). The a* value of the EWS was 4.47 and 13.2 in the 10% and 50% CIS samples, respectively, compared to the control (0.12) (Figure 7B). In the EWG, the a* value was 12.15 and 16.33 in the 10% and 50% CIS samples, respectively, compared to the control (−6.79) (Figure 7E). While the control group showed a decrease in the a* value due to the transition from EWS to EWG caused by heating, the CIS replacement group exhibited an increase in a*, resulting in enhanced redness due to gelation (Figure 7B,E). A similar trend was observed with YPF, although not as prominently as with CIS. The a* value of the BBL sample was least affected by the addition of the tannin extract (Figure 7B,E). Given that the a* value of the CIS extract was higher (Table 1), the reddish coloration of the EWS and EWG with CIS addition could be attributed to the CIS solution as a raw material.

The b* value of the EWG was 8.01 and 12.54 for the 10% and 50% CIS samples, −1.56 and 5.78 for the 10% and 50% YPF samples, and 11.39 and 14.95 for the 10% and 50% BBL samples, compared to the control (−6.72), indicating that the CIS and BBL samples produced more yellow gels (Figure 7F). The transition from EWS to EWG by heating decreased the b* value of the control from 8.43 to −6.52, whereas the BBL-added sample exhibited a remarkable increase in b* value, resulting in a more pronounced yellow color due to gelatinization (Figure 7C,F). A similar trend was noted in the CIS sample, albeit to a lesser extent than in the BBL sample. The higher b* values of the CIS and BBL solutions (Table 1) suggest that the yellowish tint of the EWG in the CIS and BBL treatments was attributed to the raw material tannin extracts.

To summarize, the BBL solution, while enhancing the yellowish color (Figure 7F), had a noticeably smaller effect on the change in the appearance of the EWG (Figure 6). Considering its effect on the change in color of the EWG, BBL stands out as a superior tannin extract material compared to CIS and YPF, due to its minimal effect on appearance (Figure 6).

### 2.6. Textural Properties and Scanning Electron Microscopy (SEM)

**Figure 8:**
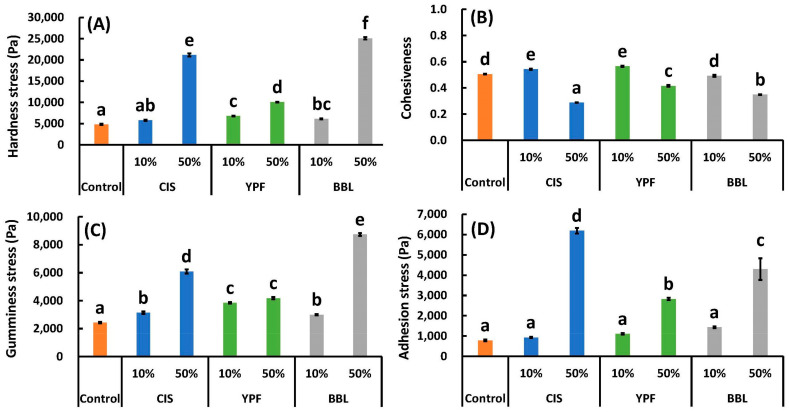
(**A**) Hardness stress, (**B**) cohesiveness, (**C**) gumminess stress, and (**D**) adhesion stress of tannin-treated egg white gels. All results were obtained using Tukey’s test for multiple comparisons. Different letters indicate significant differences at *p* < 0.05. Data are expressed as the mean ± SE (*n* = 5). CIS, chestnut inner skin; YPF, young persimmon fruit; BBL, bayberry leaf.

Figure 8 presents the results of the physical property measurements of the EWG. The hardness stress, an indicator of food hardness, was significantly higher (*p* < 0.05) in all tannin extract addition treatments than in the control, except for in the 10% CIS sample. The control registered at 4807 ± 135 Pa, while the 50% CIS sample exhibited 21,161 ± 372 Pa (4.4 times that of the control), the 50% YPF sample recorded 10,059 ± 90 Pa (2.1 times that of the control), and the 50% BBL substitution reached 25,077 ± 278 Pa (5.2 times that of the control) (Figure 8A). When tannin extracts of the same material were added, the hardness stress was significantly higher at the 50% addition amount than at the 10% amount for all CIS, YPF, and BBL samples (*p* < 0.05). These results indicate that the higher the amount of tannin extract added, the harder the EWG, with the effect being more pronounced in the order of BBL > CIS > YPF (Figure 8A).

Cohesiveness is a numerical value that indicates the percentage of elasticity remaining after chewing. When compared with the control, CIS, YPF, and BBL were equivalent to or higher than the control with a 10% addition, and the cohesiveness values were significantly lower than the control with a 50% addition (Figure 8B) (*p* < 0.05).

Gumminess stress is the product of hardness stress and cohesiveness, indicating the chewiness of the second bite. Compared to the control, all tannin extract addition treatments resulted in significantly higher gumminess stress values (*p* < 0.05). Gumminess stress was particularly high at 50% CIS and 50% BBL. The control registered at 2428 ± 63 Pa, whereas CIS 50% was 6091 ± 149 Pa, and BBL 50% was 8741 ± 102 Pa, respectively, 2.5 and 3.6 times higher than the control (Figure 8C).

The results of adhesion, indicating stickiness in the mouth, are shown in Figure 8D. The 10% tannin extract addition showed no significant difference (*p* > 0.05) compared to the control in CIS, YPF, and BBL, whereas the 50% addition showed significantly higher values (*p* < 0.05) (Figure 8D). The adhesion value of the control was 783 ± 47 Pa, whereas that of the 50% CIS sample was 6188 ± 134 Pa (7.9 times that of the control), the 50% YPF sample was 2827 ± 63 Pa (3.6 times that of the control), and the 50% BBL sample was 4297 ± 533 Pa (5.5 times that of the control). These results suggest that the addition of 50% tannin extracts makes the EWG firmer, more sticky in the mouth, and chewier the second time around.

**Figure 9:**
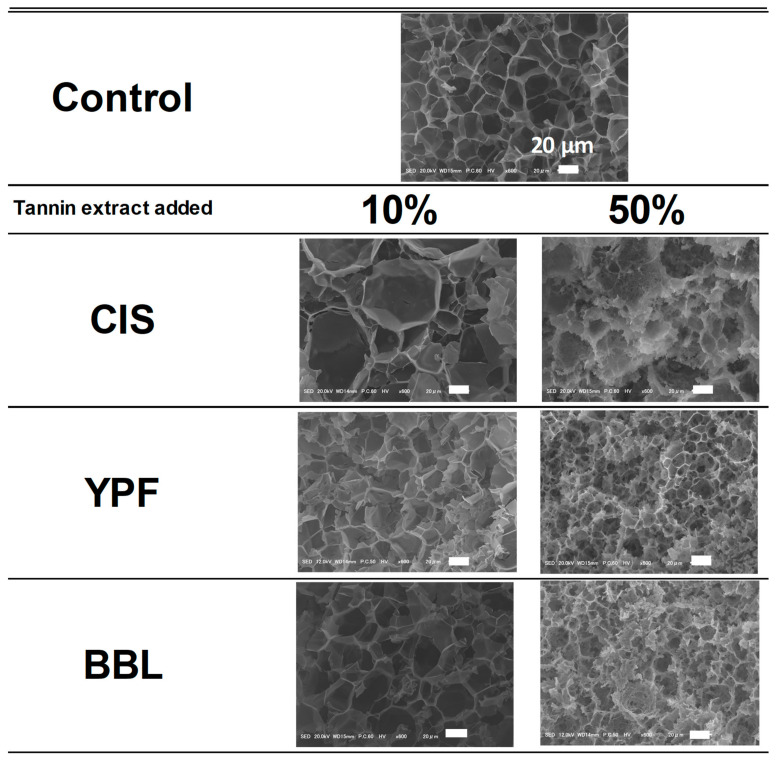
SEM image of tannin-treated EWG. SEM, scanning electron microscopy; EWG, egg white gel; CIS, chestnut inner skin; YPF, young persimmon fruit; BBL, bayberry leaf.

Next, SEM observations were made of the microstructure of the EWG, which has a crucial effect on the texture properties. In the control, a dense and regular network structure of about 20–40 µm was observed, separated by a thin film (Figure 9). When the 10% tannin extracts group was compared with the control, the network structure was larger and more varied in size in the CIS sample than in the control, while a network structure similar to the control was observed in the YPF and BBL samples. On the other hand, the addition of 50% tannin extracts showed a remarkable difference compared to the control and 10% addition samples. The membranous part forming the network was thicker with the 50% addition of both CIS, YPF, and BBL than with the control and 10% additions. Differences in tannin material were also observed; the 50% CIS addition EWG had a random network structure, as did the 10% CIS EWG addition. The EWGs with 50% YPF and 50% BBL formed denser networks compared to the respective 10% additions. The tannin material with the greatest effect on textural properties was the 50% addition of CIS and BBL, but the microstructure of the tannins was found to be different in this study. In other words, with 50% CIS addition, the films forming the network were thicker and formed a random network, whereas, with 50% BBL addition, a regular and dense network was formed. Generally, gels with a dense network become hard; however, it was not clear why the gel became strong at 50% CIS in this case.

Tsurunaga [40] prepared jelly-like confections by adding astringent persimmon or non-astringent persimmon to soy milk and found that astringent persimmon jelly had a higher fracture stress than non-astringent persimmon jelly. This difference was attributed to the complex formation between the tannins and soymilk proteins [40]. There have also been reports on the binding properties of EW proteins to tannins; Chen et al. [41] observed the characteristics of the complex of OVA and tannic acid using dynamic light scattering and interfacial tension measurements. Shen et al. [42] reported that EW with tea polyphenols decreased in α-helix content and increased in β-sheet content; Zhou et al. [43] stated that the elastic modulus of fish surimi increased as the concentration of tea polyphenols bound to EW increased. Xue et al. [44] also found that, in EW treated with tea polyphenols followed by heat treatment, the stability of the gel structure improved with increasing amounts of tea polyphenols. These results are consistent with the increase in hardness stress and gumminess stress with the addition of the tannin extract in this experiment (Figure 8A,C). Xue et al. [44] reported that the binding of tea polyphenols to EW proteins is mainly maintained by ionic and disulfide bonds. However, we considered the binding of tannins to the EW protein to be covalent based on the SDS-PAGE and FT-IR results in this study. Since the molecular weights and structures of tea polyphenols and tannins are very different, the binding mode of tannins to proteins is considered to be different.

From the above, it is suggested that the addition of tannin extracts (CIS, YPF, and BBL) can reduce allergens in EWG and make EWG harder in textural properties. In particular, the addition of the BBL solution caused little change in the appearance of the EWG, indicating that BBL was the highest-performing material in terms of EW allergen reduction effect and quality. Because tannins have an astringent taste, there is concern that their addition would alter the taste. In a sensory evaluation, we have confirmed that the astringency disappears in a small number of people. However, because the threshold for astringency varies greatly from person to person, sensory testing must be conducted for a larger number of people. Future investigation should include clinical trials on hypoallergenicity in humans and taste evaluation on a larger number of people. In the future, we hope to expand the application area of these gels by achieving hypoallergenicity while maintaining the quality of egg whites.

## 3. Materials and Methods

### 3.1. Ingredients of EW and Tannin Extract, and Preparation

The EW sample was EW-type W (52870, Kewpie Tamago Co., Tokyo, Japan) consisting of dried egg whites produced by a hygienic spray-drying method and subsequently stored in a freezer until further analysis. Egg white powder produced by the spray-drying method is widely used in industry and is of the same quality as that produced from raw eggs. Chestnut (*Castanea crenata*) inner skin (CIS), young persimmon (*Diospyros kaki*) fruit (YPF), and BBLs (*Morella rubra*) were selected for tannin extract production. To ensure the exclusive collection of the CIS, the ‘Porotan’ cultivar (Tsukuba City, Ibaraki, Japan), known for its superior peeling ability, was employed. The CIS underwent drying at 60 °C for 12 h in a constant air temperature oven (DN-61, Yamato Scientific Co., Tokyo, Japan). The YPF, sourced from the ‘Saijo’ cultivar of persimmon at the Shimane Agricultural Technology Center (Izumo City, Shimane, Japan), was freeze-dried after the removal of calyces and seeds. The BBLs, acquired from Shimane University (Matsue City, Shimane, Japan), underwent freeze-drying before use. Each sample was crushed with an Oster blender (Osaka Chemical Co., Ltd., Osaka, Japan) and passed through a 1.0 mm sieve. The processed samples were sealed in aluminum packs and stored at −25 °C.

### 3.2. Preparation of Tannin Extract

Subsequently, 45 g of the dried powder was placed in a 500 mL heat-resistant bottle, and 300 mL of distilled water was added. Hot water extraction was carried out at 120 °C for 20 min using an autoclave (LSX-300, Tommy Seiko, Tokyo, Japan). The centrifugation process at 1000× *g* for 3 min (LCX-100; Tomy Seiko Co., Tokyo, Japan) removed the powder, and the resulting supernatant served as the tannin extract.

### 3.3. EWG Production Method

The dried EW powder, weighing 5.5 g, was introduced into 94.5 g of distilled water, with constant stirring to prevent lump formation. Following the dissolution of the EW powder, the EWS was allowed to stand for 15 min. Subsequently, 15 g of the EW solution was carefully pipetted into a stainless-steel dish (40 mm × 15 mm) (ST-40, Yamaden Co., Ltd., Tokyo, Japan) to avoid bubble formation. The dish was then placed in a steam oven range (Panasonic Corporation, NE-BS1600-K, Tokyo, Japan) and heated at 90 °C for 15 min, followed by an additional 3 min of residual heat to gelatinize the product. The resultant samples were cooled in a refrigerator for 1 h before analysis. The EWGs obtained through this procedure were considered controls, lacking any tannin extract. In the tannin extract-supplemented group, either 10 (equivalent to 10% of the total weight) or 50 g (equivalent to 50% of the total weight) of distilled water was substituted with the tannin extract.

### 3.4. Evaluation of Immunoreactivity of EW Proteins

#### 3.4.1. SDS-PAGE Analysis

Sodium dodecyl sulfate-polyacrylamide gel electrophoresis (SDS) was conducted following the method outlined by Laemmli [45] with slight modifications. The sample buffer, consisting of 2-mercaptoethanol added to commercially available 2x Laemmli sample buffer (BIO RAD# 161-0737), was mixed in equal volumes with the protein solution and heated at 95 °C for 5 min. Electrophoresis utilized 10–20% e PAGEL (ATTO Co., Tokyo, Japan) and was carried out at 300 V. A total of 5.0 µL of molecular weight markers (XL-lader broad; AproScience Co., Tokushima, Japan) and the loading samples were poured into 8.5 µL wells. One-step coomassie brilliant blue (CBB) (AproScience, Tokushima, Japan) was employed for gel staining.

#### 3.4.2. ELISA

ELISA kits from the FASPEK ELISA II^®^ series for albumin (Morinaga Institute of Biological Science, Kanagawa, Japan) were employed in this study to quantify EW albumin content. ELISA was officially introduced as an analytical method in Japan in 2002 [46]. The FASPEK KIT II^®^ utilizes polyclonal antibodies to detect specific purified proteins or individual proteins of specific components, with a focus on albumin in the context of eggs [46]. Analytical procedures were conducted in accordance with the instructions provided in the kit [47], which required 19 mL of the extract to be added per gram of sample; however, in our case, the protein content derived from the antigen in the sample was substantially higher, which could lead to insufficient extraction. Therefore, we added 19.9 mL of extract to 0.1 g of the sample. The samples were appropriately diluted using specified reagents to fall within the assay range. The sample solution was added to the antibody-coated plate and allowed to stand at room temperature for 1 h. After washing, the antibody solution was added and allowed to stand at room temperature for 30 min. After the second washing operation, the enzyme–substrate solution was added and allowed to stand for 20 min at room temperature under shielded light. The absorbances were measured at 450 nm (650 nm was the reference wavelength). Four measurements were taken for each treatment group.

### 3.5. TPC and STC

The TPC was determined using the method described by Chung et al. [48], which is a modification of the Folin–Ciocalteu method [49]. The CIS, YPF, and BBL extract samples were appropriately diluted using distilled water for the TPC and STC analyses. The STC was assessed using polyvinyl polypyrrolidone (PVPP), a tannin-complexing agent [50,51]. PVPP was introduced into the extract for TPC measurements to insolubilize the tannins. The amount of PVPP (10, 20, 30, 60, 90, 120 mg) required to insolubilize the tannin was preliminarily tested, and it was confirmed that the addition of over 30 mg of PVPP to 1000 µL of the extract diluted with water showed no further insolubilization. Therefore, the amount of PVPP to be added was set at 30 mg per 1000 µL of diluted extract. The STC was then calculated by deducting the TPC value of the extract post-PVPP addition from the initial TPC value.

For the extraction of the EWG, 60% ethanol (*v*/*v*) was added to a 5.0 g sample, and homogenization was performed using a homogenizer (AHG-160D, AS ONE Co., Osaka, Japan) at 1000 rpm for 2 min. The supernatant was then centrifuged for 3 min (1000× *g*) and diluted accordingly. The TPC and STC values were expressed using catechin (CTN) as a standard, with six measurements taken for each treatment.

### 3.6. External Appearance and Color

The external appearance was captured with a digital camera (WG-40W; Ricoh, Tokyo, Japan). To determine the color of the samples, a spectrophotometer (CR-13; Konica Minolta, Tokyo, Japan) was employed. A disposable cell (optical path length 10 mm) was used to measure the color of the EWS, CIS, YPF, and BBL extracts; the EWG was measured directly on the gel surface. Each treatment underwent ten measurements.

### 3.7. SEM

The surface and cross-sectional structure of the EWG were examined through SEM. The EWG samples were cut into cubes (5 × 5 × 5 mm^3^) and fixed in a 2.5% (*v*/*v*) glutaraldehyde solution (prepared in 0.1 M, pH 7.2 phosphate buffer) for 24 h [52]. Following fixation, the samples underwent three washes with a phosphate buffer and were then lyophilized. After lyophilization, the samples were affixed to a sample stand for SEM (Nissin EM Corporation, Type-HM, Tokyo, Japan) using double-sided carbon tape (Nissin EM Corporation, 8 mm × 20 m, Tokyo, Japan). Gold deposition was carried out, and SEM observations (JSM-IT800SHL, JEOL Ltd., Tokyo, Japan) were conducted at an acceleration voltage of 20 kV with a magnification of 600×.

### 3.8. pH

The pH values of the EWS, CIS, YPF, and BBL extracts were gauged using a pH meter (LAQUA F-72, Horiba, Ltd., Kyoto, Japan). Each treatment group underwent five measurements.

### 3.9. Textural Properties

To assess the textural properties of the EWG sample in a stainless-steel dish (40 × 15 mm) (ST-40, Yamaden Co., Ltd., Tokyo, Japan), an RE2-33005B Creep Meter (Yamaden Co., Ltd., Tokyo, Japan) was employed. The measurement conditions included a 20 N load cell, SPEED 10 mm/s, and a strain rate of 66.67% with a No. 56 (φ20 mm) acrylic resin plunger attached to an L40 acrylic resin extension plunger. Measurements were conducted in a stainless-steel petri dish, and the Texture Analysis software Ver. 2.2 (Yamaden) was used to determine the hardness stress (Pa), cohesiveness, and adhesion (Pa) of each sample. Five EWGs were measured for each treatment, and presented as mean ± SE.

### 3.10. FT-IR

For the FT-IR measurements, lyophilized EWG powder was utilized to eliminate the impact of moisture [53]. FT-IR spectra were recorded on an A-Cary 630 FT-IR spectrometer (Agilent Technologies, Los Angeles, CA, USA) with a wavenumber range of 4000–400 cm^−1^ and a resolution accuracy of 2 cm^−1^ [54]. A horizontally attenuated total reflectance device (ATR) was employed to measure the spectra of the samples.

### 3.11. Statistical Analysis

Statistical analysis of the data was conducted using SPSS software (Version 28.0, SPSS, Chicago, IL, USA), and the results were expressed as mean ± SE. For multiple comparisons, data were assessed using a one-way analysis of variance (ANOVA) followed by Tukey’s test, with the significance level set at 5%.

## Figures and Tables

**Table 1:** TPC, STC, image, color, pH, and Brix data of CIS, YPF, BBL, and EWS.

	TPC	STC	Image		L*	a*	b*	pH	Brix
	(mg CTN eq/100 mL)								
CIS solution	1290.8 ± 19.2 ^c^	391.3 ± 13.3 ^b^	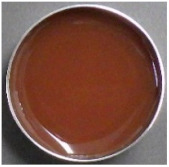	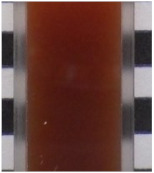	39.3 ± 0.0 ^b^	14.7 ± 0.0 ^d^	11.6 ± 0.1 ^c^	4.1 ± 0.0 ^a^	8.6 ± 0.0 ^c^
YPF solution	1868.5 ± 40.2 ^d^	331.5 ± 15.5 ^b^	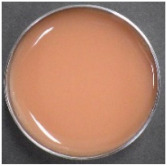	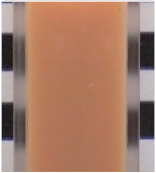	49.5 ± 0.1 ^d^	12.2 ± 0.1 ^b^	8.9 ± 0.1 ^b^	4.8 ± 0.0 ^b^	10.1 ± 0.0 ^d^
BBL solution	964.8 ± 20.7 ^b^	322.5 ± 27.9 ^b^	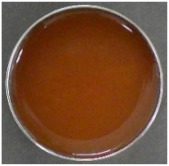	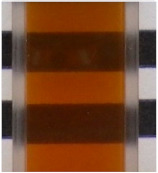	36.6 ± 0.1 ^a^	12.9 ± 0.2 ^c^	14.5 ± 0.2 ^d^	4.0 ± 0.0 ^a^	4.7 ± 0.0 ^a^
EW solution	411.5 ± 20.0 ^a^	0 ^a^	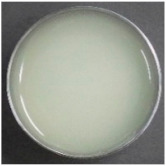	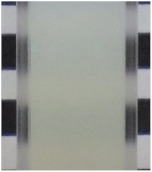	41.6 ± 0.1 ^c^	0.1 ± 0.1 ^a^	8.4 ± 0.1 ^a^	7.2 ± 0.0 ^c^	7.1 ± 0.0 ^b^

All results were obtained by Tukey’s test for multiple comparisons. Different letters indicate significant differences at *p* < 0.05. Data are expressed as mean ± SE (*n* = 6). TPC: total polyphenol content, CTN: catechin, STC: soluble tannin content, CIS: chestnut inner skin, YPF: young persimmon fruit, BBL: bayberry leaf, EW: egg white, EWS: egg white solution.

**Table 2:** Band assignment of FT-IR bands.

Wavenumber (cm^−1^)	Functional Group
1750–1700	C=O str.
1650–1480	C=C str. of aromatic rings
1400–1300	C-OH def.
1300–1160	C-OH str.
1285	C-O str. of pyran ring
1200–950	C-H def.

FT-IR, fourier transform infrared spectroscopy; str., stretching; def., deformation.

## Data Availability

Data is contained within the article.

## Abbreviations

OVM	Ovomucoid
OVA	Ovalbumin
OVT	Ovotransferrin
LYZ	Lysozyme
EW	Egg white
BBL	Bayberry leaf
EWG	EW gel
EWS	EW solution
CIS	Chestnut inner skin
YPF	Young persimmon fruit
CTN	Catechin
SE	Standard error
ANOVA	One-way analysis of variance
ATR	Attenuated total reflectance device
SEM	Scanning electron microscopy
FT-IR	Fourier transform infrared spectroscopy
TPC	Total polyphenol content
STC	Soluble tannin measurement
IR	Infrared spectroscopy
ELISA	Enzyme-Linked Immunosorbent Assay
PVPP	Polyvinyl polypyrrolidone
